# Can computer-aided diagnosis assist in the identification of prostate cancer on prostate MRI? a multi-center, multi-reader investigation

**DOI:** 10.18632/oncotarget.26100

**Published:** 2018-09-18

**Authors:** Sonia Gaur, Nathan Lay, Stephanie A. Harmon, Sreya Doddakashi, Sherif Mehralivand, Burak Argun, Tristan Barrett, Sandra Bednarova, Rossanno Girometti, Ercan Karaarslan, Ali Riza Kural, Aytekin Oto, Andrei S. Purysko, Tatjana Antic, Cristina Magi-Galluzzi, Yesim Saglican, Stefano Sioletic, Anne Y. Warren, Leonardo Bittencourt, Jurgen J. Fütterer, Rajan T. Gupta, Ismail Kabakus, Yan Mee Law, Daniel J. Margolis, Haytham Shebel, Antonio C. Westphalen, Bradford J. Wood, Peter A. Pinto, Joanna H. Shih, Peter L. Choyke, Ronald M. Summers, Baris Turkbey

**Affiliations:** ^1^ Molecular Imaging Program, National Cancer Institute, National Institutes of Health, Bethesda, MD, USA; ^2^ Imaging Biomarkers and Computer-aided Diagnosis Lab, Radiology and Imaging Sciences, Clinical Center, National Institutes of Health, Bethesda, MD, USA; ^3^ Clinical Research Directorate/ Clinical Monitoring Research Program, Leidos Biomedical Research, Inc., Frederick National Laboratory for Cancer Research, Frederick, MD, USA; ^4^ Urologic Oncology Branch, National Cancer Institute, National Institutes of Health, Bethesda, MD, USA; ^5^ Department of Urology and Pediatric Urology, University Medical Center Mainz, Mainz, Germany; ^6^ Department of Urology, Acibadem University, Istanbul, Turkey; ^7^ Department of Radiology, University of Cambridge, Cambridge, UK; ^8^ Department of Radiology, University of Udine, Udine, Italy; ^9^ Department of Radiology, Acibadem University, Istanbul, Turkey; ^10^ Department of Radiology, University of Chicago, Chicago, IL, USA; ^11^ Department of Radiology, Cleveland Clinic, Cleveland, OH, USA; ^12^ Department of Pathology, University of Chicago, Chicago, IL, USA; ^13^ Department of Pathology, Cleveland Clinic, Cleveland, OH, USA; ^14^ Department of Pathology, Acibadem University, Istanbul, Turkey; ^15^ Department of Pathology, University of Udine, Udine, Italy; ^16^ Department of Pathology, University of Cambridge, Cambridge, UK; ^17^ Department of Radiology, Federal Fluminense University, Rio de Janeiro, Brazil; ^18^ Department of Radiology, Radboud University, Nijmegen, The Netherlands; ^19^ Department of Radiology, Duke University, Durham, NC, USA; ^20^ Department of Radiology, Hacettepe University, Ankara, Turkey; ^21^ Department of Radiology, Singapore General Hospital, Singapore; ^22^ Weill Cornell Imaging, Cornell University, New York, NY, USA; ^23^ Department of Radiology, Mansoura University, Mansoura, Egypt; ^24^ UCSF Department of Radiology, University of California-San Francisco, San Francisco, CA, USA; ^25^ Center for Interventional Oncology, Clinical Center, National Institutes of Health, Bethesda, MD, USA; ^26^ Biometric Research Branch, National Cancer Institute, National Institutes of Health, Bethesda, MD, USA

**Keywords:** computer-aided diagnosis, prostate cancer, multiparametric MRI, PI-RADSv2, tumor detection

## Abstract

For prostate cancer detection on prostate multiparametric MRI (mpMRI), the Prostate Imaging-Reporting and Data System version 2 (PI-RADSv2) and computer-aided diagnosis (CAD) systems aim to widely improve standardization across radiologists and centers. Our goal was to evaluate CAD assistance in prostate cancer detection compared with conventional mpMRI interpretation in a diverse dataset acquired from five institutions tested by nine readers of varying experience levels, in total representing 14 globally spread institutions.

Index lesion sensitivities of mpMRI-alone were 79% (whole prostate (WP)), 84% (peripheral zone (PZ)), 71% (transition zone (TZ)), similar to CAD at 76% (WP, p=0.39), 77% (PZ, p=0.07), 79% (TZ, p=0.15). Greatest CAD benefit was in TZ for moderately-experienced readers at PI-RADSv2 <3 (84% vs mpMRI-alone 67%, p=0.055). Detection agreement was unchanged but CAD-assisted read times improved (4.6 vs 3.4 minutes, p<0.001). At PI-RADSv2 ≥ 3, CAD improved patient-level specificity (72%) compared to mpMRI-alone (45%, p<0.001).

PI-RADSv2 and CAD-assisted mpMRI interpretations have similar sensitivities across multiple sites and readers while CAD has potential to improve specificity and moderately-experienced radiologists’ detection of more difficult tumors in the center of the gland. The multi-institutional evidence provided is essential to future prostate MRI and CAD development.

## INTRODUCTION

Men with suspected or known prostate cancer are increasingly evaluated with prostate multiparametric MRI (mpMRI) because it aids in the detection of clinically significant disease [1–4]. However, mpMRI has been criticized because of variability in quality of exams and inconsistent interpretations across clinical centers and physicians. Interpretation can be affected by many factors including relative visibility of tumors, tumor location, and inter-observer variation. To address some of these issues the Prostate Imaging-Reporting and Data System version 2 (PI-RADSv2) was introduced in 2015 as a set of guidelines outlining standard acquisition parameters and a categorization system for cancer detection [5]. PI-RADSv2 has been widely adopted and can achieve cancer detection rates up to 80-90%; however, it is associated with a steep learning curve and exhibits a high degree of inter-reader variability, likely reflecting inherent ambiguities in the classification scheme. Moreover, many centers report a mpMRI miss rate up to 16-30% [6–11]. A large scale, prospective, multicenter trial of PI-RADSv2 has not yet been performed.

Machine learning is a highly touted method of improving feature recognition in images. Computer-aided diagnosis (CAD) systems have shown promise in the identification of prostate cancer on mpMRI in several single institution studies [12–17]. For instance, we previously developed a CAD system based on in-house images with readers from outside our institution and showed excellent results [18]. However, for a CAD system to be truly useful it must be trained with a much more diverse set of data, crossing vendors and institutions, and interpreted by multiple readers with varying experience to validate its performance.

The purpose of this study was to test a new prostate CAD on a highly heterogenous, “real-world” data set from 5 institutions against mpMRI interpretations with PI-RADSv2 using a diverse set of readers, varying in location and experience.

## RESULTS

### Patient and lesion characteristics

Patient and lesion characteristics are given in Table 1, stratified by institution. The final study population consisted of 144 case patients and 72 control patients. In case patients, there were a total of 285 pathologically-proven tumors, of which 10/285 were found spanning both peripheral (PZ) and transition (TZ) zones, 187/285 were PZ lesions, and 88/285 were in the TZ. Institution 1 had the highest proportion of Gleason score 3+3, at 57% (27/47), whereas Institutions 2, 3, and 5 most commonly reported Gleason 7.

**Table 1:** Patient and tumor demographics by providing institution

		Institution 1		Institution 2		Institution 3		Institution 4	Institution 5	Total		
		Cases	Controls	Cases	Controls	Cases	Controls	Cases	Cases	Cases	Controls	*p*
Patient-based	N	32	24	36	24	50	24	10	16	144	72	
	Age	65.6 (51-76)	61.3 (49-78)	61.8 (51-71)	59.9 (49-72)	61.9 (47-79)	62.8 (50-77)	58.5 (42-68)	63.1 (54-76)	62.6 (42-79)	61.3 (49-78)	*0.21*
	PSA	8.4 (3.3-23)	10.9 (3.5-24)	9.3 (3.4-26.1)	6.6 (0.3-11.5)	6.7 (1.2-27.3)	6.9 (1.3-24)	11 (3.7-31.9)	7.5 (3.5-17.8)	8.1 (1.2-31.9)	8.2 (0.3-24)	*0.5*
	Mean # lesions/ patient	1.47		2.06		1.98		3.3	2	1.98		

		WP	PZ	TZ	WP	PZ	TZ	WP	PZ	TZ	WP	PZ	TZ	WP	PZ	TZ	WP	PZ	TZ	
Lesion-based by Gleason score	Total	47	35	14	74	54	22	99	65	38	33	19	16	32	24	8	285	197^*^	98^*^	
	3+3	27	19	8	18	9	9	10	6	4	17	8	9	0	0	0	72	42	30	
	3+4	9	6	3	42	34	8	64	42	23	11	6	5	19	17	2	145	105	41	
	4+3	5	5	1	5	5	2	14	7	8	3	3	1	10	5	5	37	25	17	
	4+4	3	3	0	5	4	1	2	2	0	0	0	0	0	0	0	10	9	1	
	>4+4	3	2	2	4	2	2	9	8	3	2	2	1	3	2	1	21	16	9	

Lesion-based data is given for whole prostate (WP) and by zone (peripheral (PZ), transition (TZ)). ^*^10 lesions located in both PZ and TZ.

### Patient based baseline mpMRI and CAD performance

The areas under the receiver operating characteristic curves (AUCs) of mpMRI alone (mpMRI) and CAD-assisted mpMRI interpretation (CAD) for the detection of cancer at the patient-level were 81.9% and and 83.1%, respectively (p=0.58).

Patient-level sensitivity and specificity of mpMRI and CAD at each PI-RADSv2 category threshold is given in Table 2. Considering all detected lesions (PI-RADSv2 category ≥1), moderately experienced readers achieved comparable sensitivity with mpMRI vs CAD at 93.3% vs 92.8% (p=0.864) while highly experienced readers achieved mpMRI sensitivity 96.7% vs CAD at 90.7% (p=0.007). Overall specificity was similar between mpMRI and CAD, at 35% for both (p=0.927).

**Table 2:** Patient-level sensitivity and specificity of mpMRI and CAD at each PI-RADSv2 category threshold

		Overall			Moderately experienced			Highly experienced		
PI-RADSv2 Threshold		MRI	CAD	*p*	MRI	CAD	*p*	MRI	CAD	*p*
1	Sensitivity	95.6% *(92.9-97.7%)*	91.4% *(86.5-94.4%)*	*0.05*	93.3% *(88.7-97.1%)*	92.8% *(87.3-97.2%)*	*0.86*	96.7% *(94.5-98.5%)*	90.7% *(85.3-95.3%)*	*0.007*
	Specificity	35% *(27.6-42.6%)*	34.5% *(23.2-46.4%)*	*0.93*	44.9% *(34.2-55.6%)*	23.8% *(11.9-36.8%)*	*0.003*	30.1% *(21.2-39.1%)*	39.9% *(27.3-53.1%)*	*0.10*
2	Sensitivity	95.6% *(92.9-97.7%)*	85.4% *(79.8-91%)*	*<0.001*	93.3% *(88.7-97.1%)*	84.8% *(77.4-91%)*	*0.005*	96.7% *(94.5-98.5%)*	85.8% *(79.7-91.6%)*	*<0.001*
	Specificity	35.9% *(28.6-43.3%)*	52.1% *(42.6-62%)*	*<0.001*	44.9% *(34.2-55.6%)*	46.3% *(33.6-59.2%)*	*0.85*	31.4% *(23-39.8%)*	55% *(45.1-65.6%)*	*<0.001*
3	Sensitivity	93.9% *(90.8-96.4%)*	81.5% *(75-87.6%)*	*<0.001*	92.7% *(88.1-96.6%)*	79.4% *(71.4-86.9%)*	*<0.001*	94.4% *(91.2-97.1%)*	82.5% *(75.9-89%)*	*<0.001*
	Specificity	44.8% *(37.7-51.9%)*	71.5% *(63.2-79.6%)*	*<0.001*	48.9% *(38.1-59.6%)*	71.1% *(58.9-82.6%)*	*0.001*	42.8% *(35-50.9%)*	71.7% *(62.6-80.3%)*	*<0.001*
4	Sensitivity	88.1% *(83.6-92.3%)*	76.5% *(69.6-82.8%)*	*<0.001*	85.4% *(79.5-90.8%)*	74.1% *(65.8-82%)*	*0.001*	89.5% *(84.4-93.9%)*	77.7% *(70.5-84.4%)*	*<0.001*
	Specificity	61.9% *(53.5-69.7%)*	85.2% *(78.2-91.1%)*	*<0.001*	58% *(45.3-70.3%)*	81.5% *(70-91.4%)*	*<0.001*	63.8% *(55.1-72.5%)*	87% *(81-92.7%)*	*<0.001*
5	Sensitivity	47.7% *(40.1-55.6%)*	43.4% *(35.7-51.3%)*	*0.03*	49.8% *(39.8-60%)*	44.9% *(35.2-54.6%)*	*0.16*	46.7% *(39-54.8%)*	42.7% *(35-50.8%)*	*0.07*
	Specificity	92.7% *(89.9-95.5%)*	96.5% *(93.6-98.8%)*	*0.04*	88.4% *(82-94.7%)*	96% *(89.4-100%)*	*0.09*	94.9% *(91.4-97.9%)*	96.8% *(93.8-99.3%)*	*0.36*

For each PI-RADv2 category threshold, sensitivity and specificity are given across all readers and stratified by experience, with 95% confidence intervals given in parentheses. p<0.05 was used for significance.

When considering those lesions classified as suspicious (PI-RADSv2 category ≥3), the specificity of CAD was higher than that for mpMRI (71.5% vs 44.8% for all readers, p<0.001), while the sensitivities were comparable across reader experience levels. On mpMRI alone, highly experienced readers achieved sensitivity of 94.4% vs 92.7% for moderately experienced readers; for CAD, highly experienced readers achieved sensitivity of 82.5% vs 79.4% for moderately experienced readers.

### Lesion based PI-RADSv2 performance

**Figure 1:**
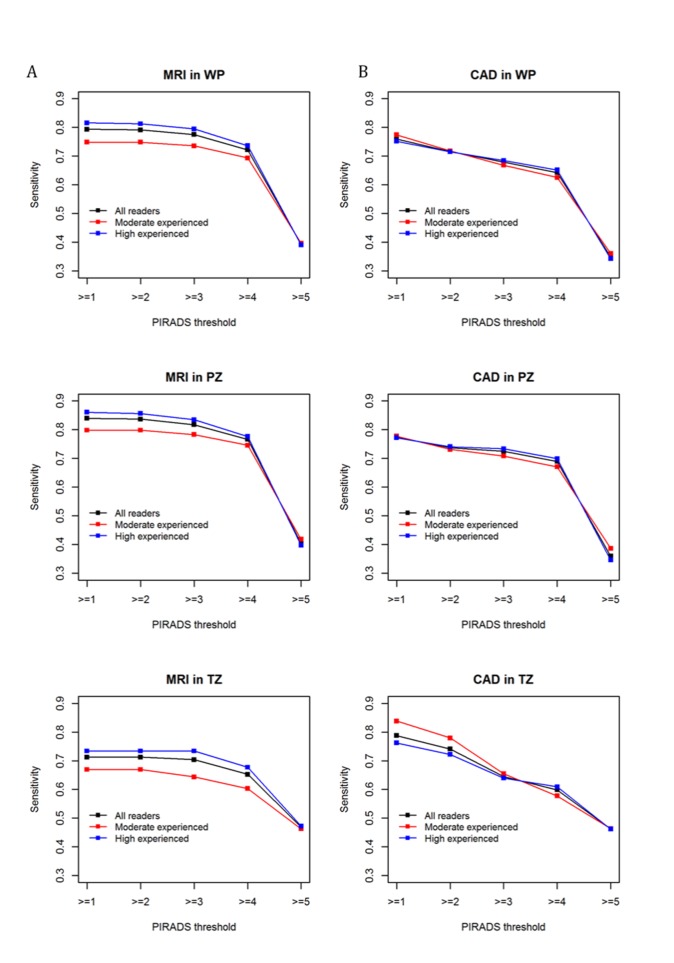
Index lesion sensitivity in WP, PZ, TZ for MRI-only **(A)** and CAD-assisted **(B)** reads. Sensitivities are plotted for all readers as well as by experience level at each PI-RADSv2 category threshold. PI-RADSv2 category ≥1 threshold used for all lesions detected on MRI and CAD, while PI-RADSv2 category ≥3 threshold used to represent all lesions considered cumulatively suspicious on MRI and CAD. WP = whole prostate, PZ = peripheral zone, TZ = transition zone.

MRI-only index lesion sensitivity stratified by reader experience at each PI-RADSv2 category threshold is given for WP, PZ, and TZ in Figure 1A. At PI-RADSv2 ≥ 1, mpMRI sensitivity for all readers was 79% (75% for moderately experienced readers and 82% for highly experienced readers). For PI-RADSv2 ≥ 3, it was similar at 78% for all readers, 74% for moderately experienced readers, and 79% for highly experienced readers.

In the PZ, mpMRI sensitivity at PI-RADSv2 ≥ 1 for all readers was 84%, and at PI-RADSv2 ≥ 3 sensitivity was 82%. Stratified by experience, moderately experienced readers achieved 80% sensitivity at PI-RADSv2 ≥ 1 and 78% at PI-RADSv2 ≥ 3 in the PZ, while highly experienced readers achieved 86% at PI-RADSv2 ≥ 1 and 83% at PI-RADSv2 ≥ 3.

In the TZ, mpMRI sensitivity at PI-RADSv2 ≥ 1 was 71% across all readers and 70% across all readers at PI-RADSv2 ≥ 3. Stratification by experience revealed that while highly experienced readers achieved sensitivities of 73% at both PI-RADSv2 thresholds, moderately experienced readers had 67% sensitivity at PI-RADSv2 ≥ 1, decreased to 64% at PI-RADSv2 ≥ 3.

### Lesion based CAD-assisted performance

CAD-assisted index lesion sensitivity stratified by reader experience is given for WP, PZ, and TZ in Figure 1B. CAD performance followed a similar trend to mpMRI-only PI-RADSv2 performance for readers of all experience, except achieving a higher detection sensitivity in the TZ at lower PI-RADSv2 categorization for moderately experienced readers.

In WP, CAD-assisted reader sensitivity at PI-RADSv2 ≥ 1 was 75% across all 9 readers, decreasing to 67.9% at PI-RADSv2 ≥ 3. Stratification by experience revealed the same trend, with moderately experienced readers achieving 77.4% sensitivity at PI-RADSv2 ≥ 1 but 66.8% at PI-RADSv2 ≥ 3 and highly experienced readers achieving sensitivities of 75.2% vs 68.5% for PI-RADSv2 ≥ 1 vs ≥ 3, respectively.

In zone-based analysis, PZ CAD performance was similar to the WP. Moderately experienced readers and highly experienced readers achieved similar sensitivities at PI-RADSv2 ≥ 1, at 77.7% and 77.2%, respectively. At PI-RADSv2 ≥ 3, highly experienced readers achieved a slightly higher sensitivity than moderately experienced readers, at 73.4% vs 70.8%, respectively.

The use of CAD in the TZ demonstrated better performance for baseline detection at PI-RADSv2 ≥1, at 83.5% for moderately experienced readers and 76.2% for highly experienced readers. Performance at PI-RADSv2 ≥ 3 showed a similar trend to WP and PZ, with sensitivity 65.5% for moderately experienced readers and 64% for highly experienced readers.

### Lesion based comparison

**Figure 2:**
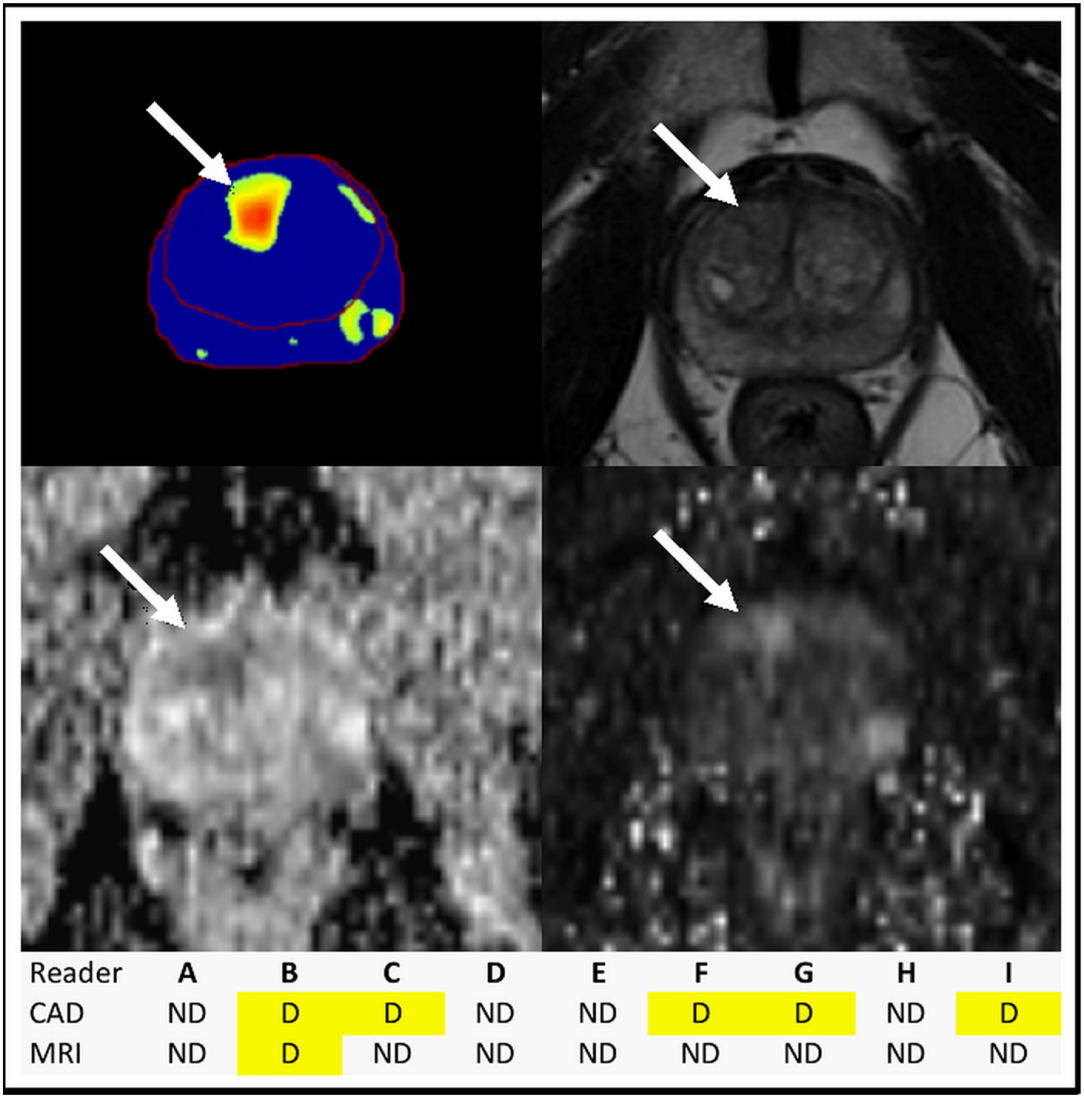
Benefit of CAD in TZ tumor identification CAD (top left) picked up a tumor (arrows) in the right apical anterior TZ, identified by more readers on MRI (T2W top right, ADC map bottom left, b-1500 bottom right) with CAD assistance. ND = not detected, D = detected; the tumor was found by 5 readers with CAD assistance versus 1 reader with mpMRI alone. Radical prostatectomy histopathology mapping revealed Gleason 4+5 prostatic adenocarcinoma within this lesion.

Averaged across all readers at PI-RADSv2 ≥ 1, CAD showed similar index lesion sensitivities to mpMRI-alone in WP (p=0.39), PZ (p=0.07) and TZ (p=0.15). Notably for moderately experienced readers the CAD showed a trend toward improved sensitivity in the TZ compared to mpMRI-alone for PI-RADSv2 ≥1 (83.8% CAD vs 66.9% MRI, p=0.055). Figure 2 shows an example of how CAD can benefit the detection of a TZ lesion. Similar results were observed for clinically significant cancer detection as shown in Supplementary Figure 1. In the WP, CAD lesion detection at PI-RADSv2 ≥ 1 was comparable to mpMRI-alone within experience levels (moderate: p=0.63, high: p=0.09); in the PZ, CAD detection was comparable to mpMRI-alone for moderately experienced readers (p=0.646) but not for highly experienced readers (p=0.02).

Investigation of image quality in the data set is reported in Supplementary Materials. Across all institutions’ images, 24% (35/144) of case patients and 24% (17/72) of control patients were classified as low quality based qualitative criteria of motion and rectal gas. CAD sensitivity at PI-RADSv2 ≥ 1 did not vary between high quality and low quality images at 54.6% vs 51.5%. However, fewer false positives were seen in higher quality images compared to lower quality images, resulting in CAD-assisted positive predictive value (PPV) difference of 9.4% at PI-RADSv2 ≥ 1 (57.6% for high quality vs 48.2% for low quality) and 7.5% at PI-RADSv2 ≥ 3 (76.8% for high quality vs 69.7% for low quality).

### Reader agreement

Reader agreement for lesion detection is given in Table 3 , stratified by reader experience levels. Overall agreement was not different with CAD assistance (mpMRI-alone index of specific agreement (ISA) 90% vs CAD-assisted 92%, p=0.401), and this pattern was consistent across comparisons between readers of each experience level.

**Table 3:** Inter-reader agreement of lesion detection

Reader experience level pairing	MRI	CAD	*p*
Overall	92% *(86.9-95.8%)*	89.8% *(83.7-94.9%)*	*0.401*
High-High	92.2% *(86.4-96.3%)*	88.7% *(81.7-94.7%)*	*0.251*
Moderate-Moderate	91.7% *(84-97.3%)*	91.9% *(84.5-97.4%)*	*0.963*
High-Moderate	92% *(86.8-95.6%)*	90.5% *(84.4-95.2%)*	*0.563*

**Inter-reader agreement, measured with index of specific agreement (ISA), is given across all reads and between readers of each experience level, with 95% confidence intervals given in parentheses. A p-value <0.05 was used for significance.**

### Image interpretation times

For all readers, the average time to interpret mpMRI alone and with CAD assistance was 4.6 minutes and 3.7 minutes, respectively (p<0.001). Both moderately experienced readers and highly experienced readers had reduced read out times with CAD assistance compared to mpMRI alone (moderate: mpMRI 6.3 minutes vs CAD assisted 4.4 minutes; high: mpMRI 3.5 minutes vs CAD assisted 2.7 minutes, p<0.001).

## DISCUSSION

Using moderately and highly experienced readers we observed that standardized PI-RADSv2 categorization and our CAD system, optimized for quantitative parameters that can be extracted from images obtained across different manufacturers and institutions, showed similar baseline detection rates. The CAD system additionally demonstrated improved specificity in conjunction with PI-RADSv2 categorization as well as slightly improved radiologist efficiency. Our findings suggest that standardization and interpretive assistance strategies such as PI-RADSv2 and CAD systems help readers detect cancer with reasonable accuracy, and CAD has potential to improve detection performance in the TZ. This is a robust result based on multi institutional data and a multi-reader study with non-overlapping affiliations.

PI-RADSv2 was released to promote global standardization of mpMRI and to diminish variation in acquisition, interpretation, and reporting [5]. As it is a system based on mostly expert consensus, there have been numerous post hoc studies validating PI-RADSv2. A recent meta-analysis published by Zhang et al. found a pooled sensitivity of 85% (range 78-91%) across 13 studies individually utilizing imaging data within their own institutions compared to radical prostatectomy specimens [19]. This is in agreement with other studies conducted at a single center but with multiple readers [6, 20]. In our study, we found an index lesion sensitivity for PI-RADSv2 ≥ 3 of 78%. PI-RADSv2’s intended aim is to broadly standardize interpretation; our findings largely support this aim.

A known weakness of mpMRI is the TZ where sensitivity is particularly low. The TZ is difficult because of its complex, variable architecture where features of cancer overlap with prostatic hyperplasia [21, 22]. Interestingly, for lesions scored PI-RADSv2 ≥1, the greatest CAD benefit was seen in the TZ where it helped moderately experienced readers to achieve 83.8% sensitivity with CAD versus 66.9% with mpMRI. Thus far, CAD has shown promise in PZ tumor detection but poor diagnostic value in the TZ [17, 23, 24]. Our CAD system utilized separate TZ segmentation and was precisely trained on TZ tumor outlines which may account for the unexpectedly good results which held up even at a multi-institutional level. The numerous additional true positive lesions identified with lower PI-RADS scores suggest that perhaps CAD can provide a special utility in identifying these difficult-to-see TZ tumors, especially for less experienced readers. Additionally, the classification of these tumors to PI-RADS 1 and 2 supports growing evidence that current PI-RADSv2 TZ criteria does not fully account for the spectrum of lesions encountered [25, 26].

Reader experience influenced the value of CAD compared to mpMRI alone at threshold PI-RADSv2 category ≥ 1 versus category ≥ 3. A threshold set at PI-RADSv2 category ≥ 1 represents all subjectively high probability spots indicated by the CAD alone without radiologist discretion in assigning a final suspicion score. Here, CAD in isolation demonstrated comparable performance to mpMRI alone. However, when CAD and the radiologist were considered a diagnostic team, fewer true positive lesions were identified as cumulatively suspicious i.e. PI-RADSv2 category ≥ 3 while per-patient specificity improved, especially for more experienced readers. One possible explanation for this decrease in sensitivity is variable trust in the CAD among readers. Trust has been identified as a major factor in reducing the effectiveness of CAD in radiology [27]. Distrust of CAD is more common in radiologists who are independently confident in lesion identification, and leads to under-reliance and subsequent misclassification of true positive lesions [28, 29]. Alternatively, over-reliance on CAD occurs when readers are uncertain and welcome the assistance of the CAD [30]. The prior first-reader CAD study we conducted also saw greater benefit at a PI-RADSv2 ≥ 1 threshold [18]. While neither study was designed for this purpose, the consistent pattern supports a complex CAD-user relationship which should inform future studies.

It should be noted that the cases used in this study were very diverse in terms of institution-specific acquisition, MRI manufacturer, and patient population. Single-institution studies designed for parallel training and testing of prostate CAD have reported AUCs ranging 76-95% on 3-Tesla images with biopsy or prostatectomy validation [12, 17, 31]. However, a reasonable concern in CAD development is whether a system can be used beyond the population it is trained to recognize patterns on. In our study, we observed AUC of 83% in validation of our CAD that was naïve to the variety of machine specifications and institutional protocols representative of the testing population. This demonstrates that a system trained on quantitative parameters extracted from standardized images can provide meaningful interpretive assistance in a diverse, real-world clinical application.

One potential explanation for the varying mpMRI-CAD discrepancies was image quality. It is widely acknowledged that prostate mpMRI is technically challenging due to motion artifact and rectal gas causing susceptibility artifacts on diffusion weighted imaging. Previously, Caglic et al. demonstrated a 17.5% reduction in positive predictive value where there was greater rectal gas distention. A significant negative correlation between rectal distension and DWI or T2W image quality has been noted [32]. In our population, 24% of cases had poor quality images based on evaluation of rectal distension and inter-slice prostate motion, and CAD pick-ups were more likely to be false positives in these patients resulting in a 7-10% positive predictive value reduction compared to image quality judged to be satisfactory. These issues are not helped by the wide variability in technique that has been observed in surveys of practices performing mpMRI. Esses et al. conducted a survey that revealed a highly variable level of adherence to PI-RADSv2 technical standards across imaging facilities, suggesting that image quality may often be compromised [33]. This problem can only be overcome by better training and education. However, to expect a CAD system to perform equally well on non-standard image acquisitions is unrealistic.

Our study has several limitations. First, a multi-institutional data set inherently suffers from incomplete standardization across institutions in imaging and in histopathology. This includes controls, who had negative imaging validated by 12 or 24-core biopsy. However, all institutions providing images and data were large centers with a genitourinary focus in both their radiology and pathology departments, and the natural variation seen among institutions aligned with the goal of this study to mimic real world clinical variability. Additionally, while the first-reader design most clearly elucidates contribution of CAD to final detection performance, its biggest limitation is that it does not capture additional reader pick-ups on the mpMRI. Studies in other organ systems have found that strict CAD-based decision thresholds, such as a focused probability map, may lead to less of an effort in identifying other abnormalities [34]. Alternatives include a second-reader study design in which CAD output is only available as an adjunct after the reader has viewed the images, or a CAD providing less specific prompting by drawing attention to general suspicious regions of the prostate rather than fully delineating a lesion. Another limitation of this study is that the institutions in this study were likely to be more experienced in mpMRI than an average center. Indeed, there were no inexperienced readers in the study. The readers were, in general, motivated, academic faculty which is unlikely to represent the novice general reader for which the benefits of CAD may be more striking. However, this is a fundamental limitation of clinical trials that are often first reported in academic settings. Finally, the training population utilized for our CAD system was relatively limited in an effort to prevent overlap with the study population. It has previously been shown that CAD sensitivity can be increased with a larger fraction of difficult cases included in the training database [35]. A focus on further diversifying the training population might improve the results of CAD validation.

In conclusion, when using PI-RADSv2 and a CAD system based on heterogeneous imaging acquisitions, readers with different experience levels were able to detect index lesions with comparable sensitivity to non-assisted interpretations. The addition of CAD improved reader specificity and provided a time efficiency advantage. In order to be robust, CAD systems must be based on diverse data sets and be tested by multiple readers with varying experience and diversity of location.

## MATERIALS AND METHODS

This Health Insurance Portability and Accountability Act-compliant retrospective evaluation of prospectively acquired multi-institutional data was approved by our local ethics committee. Inclusion of outside institution anonymized data was approved in accordance with the National Institutes of Health’s Office of Human Subjects Resources protocol (Protocol #11617). Local ethics approvals to share data were obtained as needed.

### Study design and statistical powering

**Figure 3:**
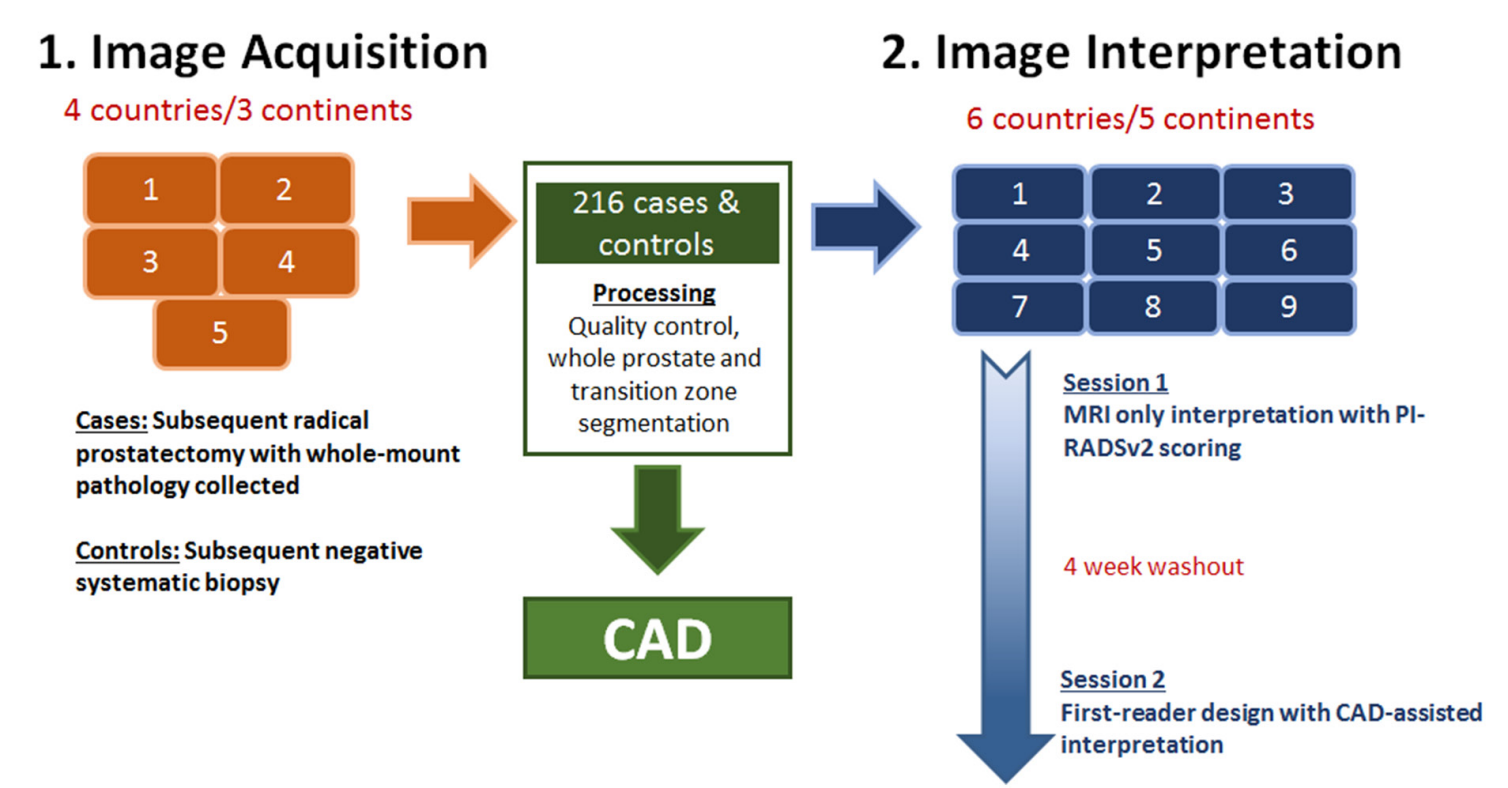
Study design The large multi-institutional framework is shown starting with image acquisition and ending with image interpretation across multiple institutions and readers.

A flow diagram illustrating overall study design is given in Figure 3. Our goal was to test PI-RADSv2 interpretation and CAD-assisted interpretation on a large scale, utilizing 5 institutions for image acquisition and 9 different institutions for image interpretation thus ensuring no local bias associated with interpreting images from one’s own institution. Our primary hypothesis was that CAD-assisted mpMRI would have a higher sensitivity for cancer detection than mpMRI alone. To limit the number of cases each reader would have to interpret, a hybrid design was used to test this hypothesis. Randomization stratified by patient disease status was carried out such that one sixth of randomly selected patients were evaluated by all readers and the remaining patients were assigned at random to each pairwise combination of readers. With this design the average number of interpretations was 76 (range 75-78) with a 2:1 ratio of cancer vs control patients for each reader. The primary endpoint was the difference in average reader-specific sensitivity between CAD and mpMRI. Based on prior results, sensitivity for index lesions was set at 76% for mpMRI, and an improvement in sensitivity of 10% was targeted for CAD [18]. The standard deviation of the endpoint was estimated using these two sensitivity values. The study has 91% power to detect a 10% difference in sensitivity using the Z test at the two-sided 5% significance level.

### Patient population

Five institutions were recruited to submit de-identified data of consecutive patients who underwent prostate mpMRI at 3-Tesla without endorectal coil that met specified standard sequences as described below. Case patients were consecutive men with lesions detected on mpMRI, subsequent positive biopsy and radical prostatectomy, and whole-mount pathology with lesion mapping available. Control patients were those with negative mpMRI and subsequent negative standard 12-core systematic biopsy or 24-core transperineal template biopsy. Patients who received any prior treatment, with imaging artifact arising from hip prostheses, or with incomplete mpMRI scans were excluded. A total of 144 case patients and 72 control patients were included. Patient characteristics are given in Table 1.

### MRI technique

All prostate mpMRI scans were acquired on 3T scanners without the use of an endorectal coil. Magnet brands and models, as well as scanning parameters, varied, but all protocols included axial, sagittal, and coronal T2 spin echo sequences without fat suppression, diffusion-weighted imaging (DWI) images acquired with at least 2 *b-*values to allow for calculation of apparent diffusion coefficient (ADC) maps and a high value *b-*1500 DWI, and unprocessed dynamic contrast enhanced (DCE) images compliant with PI-RADSv2 standards.

Supplementary Tables 1-5 contain sequences, coil information, and MRI acquisition parameters utilized in this study. For subsequent CAD processing, a high *b-*value image of b=1500 mm/sec^2^ was needed, and so in cases where this was not available, the high b value image was calculated utilizing the mono-exponential model [36].

### Image de-identification

To comply with the Office of Human Subjects Resources guidelines for utilization of external data, all images had to be completely de-identified at their respective original institutions prior to collection to ensure patient confidentiality. This de-identification was performed using standard scripts removing patient information as well as clearing DICOM tags other than those reflecting scanner parameters. Upon our collection of the data, an additional de-identification script was used to immediately process the images for a second time to guarantee patient privacy.

### Radiologist profile

Nine radiologists, from 9 different institutions, participated in the study. Six were highly experienced (>2000 prostate mpMRI cases) and three were moderately experienced (500-1000 cases). All had experience with PI-RADSv2 at their home institutions prior to this study, but none had interpreted studies from the institutions providing the images.

### Computer aided diagnosis software

The CAD system was closely based on a Random Forest classifier system developed and validated for in-house images acquired at 3T with endorectal coil [37]. The system was re-designed for optimal processing of non-endorectal coil images. T2W, ADC, *b-*1500 DWI, and segmentations of the whole prostate and transition zone were inputs for both training and study data. DCE data was not incorporated into the CAD system. Commercial software was used for automated segmentation on axial T2W images (iCAD, Nashua, New Hampshire), and each automated segmentation was further refined by a prostate mpMRI-focused research fellow with experience in segmenting >200 axial T2W prostate scans. The T2W segmentation was also used on ADC and *b-*1500 DWI images, as minimal motion was assumed between the consecutively obtained sequences. The classifier was trained based on specific tumor segmentations in a training population correlating with pathologic data from whole mount pathology, given in Supplementary Table 6. The training and study data sets had no patient or institutional overlap.

### Image interpretation

For each sequential interpretation session, readers were provided their respective assigned patients as full sets of de-identified DICOM images and instructed to view them on their personal workstations utilizing RadiAnt DICOM Viewer [38]. Readers were unaware of clinical and pathologic outcomes.

For Session 1, the sequences provided for each patient consisted of T2W, DWI (ADC, *b-*1500 DWI), and DCE. Through a Microsoft Access-designed read-out form, each reader was provided with patient pseudo-identifiers in a randomized order. Within this programmed form, readers recorded up to 4 detected lesions per mpMRI, assigned a PI-RADSv2 category from 1-5 to each lesion, recorded the location of each lesion in a standardized fashion that included the zone, side of the prostate, and an annotated screenshot of the lesion [5]. Additionally, the form was built with a timer that recorded duration of each interpretation session. All data were recorded in a linked Microsoft Access database [39].

A 4-week washout period followed the conclusion of Session 1. During the washout, a training packet was sent to each reader with 3 examples to familiarize them with interpreting the CAD results. For Session 2, readers were instructed to view the CAD output first and identify up to 4 suspicious areas which were markedly red or orange on a probability map as shown in Supplementary Figure 2. Following CAD output interpretation, readers evaluated the full corresponding mpMRI next to the annotated CAD output. Readers accepted the finding on CAD if mpMRI features were consistent with PI-RADSv2 category ≥ 3; otherwise the finding on CAD was rejected (PI-RADSv2 category ≤ 2) [5]. Each patient was assigned a new pseudo-identifier and the patient list was randomized from Session 1 to ensure that studies were interpreted in a different order. Readers input data on a similar Microsoft Access form that recorded time and linked the data to the database [39]. For both sessions, PI-RADSv2 ≥ 1 represents all recorded lesions, and PI-RADSv2 ≥ 3 represents those lesions classified as appearing more suspicious according to PI-RADSv2 guidelines [5].

### Histopathologic validation

Each providing institution was instructed to supply mapped histopathology of the radical prostatectomy specimen spanning from apex to base. Lesion-specific locations and Gleason scores were determined by a genitourinary pathologist from each institution. Pathologists were unaware of mpMRI results. MRI-histopathology correlation was performed by a prostate mpMRI-focused research fellow using visible prostate landmarks and lesion morphology.

### Statistical analysis

For patient-based analysis, the maximum PI-RADSv2 score assigned by a given reader was used to calculate the sensitivity and specificity at each PI-RADSv2 threshold and to construct a receiver operating characteristic (ROC) curve. For lesion-based analysis, reader sensitivity for index lesions was calculated. The index lesion was defined as the tumor with highest Gleason score and largest volume as designated on histopathology. Specificity was not estimated because negative regions were not specified. Because true positive lesions detected by CAD but rejected by readers would be assigned PI-RADSv2 categories 1 or 2, the comparison in true positives between CAD and mpMRI alone was focused on PI-RADSv2 ≥ 1 and PI-RADSv2 ≥ 3. Reader statistics were averaged across all readers and by experience level. Bootstrap resampling stratified by disease status was used to calculate the 95% confidence intervals for sensitivity, specificity, and area under the ROC curve (AUC). The confidence limits were obtained from the 2.5^th^ and 97.5^th^ percentiles of the 2,000 bootstrap samples. The Wald test using the bootstrap standard error was utilized to test the differences in the estimated sensitivity, specificity, and AUC between mpMRI and CAD. All tests were two-sided and p-value <0.05 was considered statistically significant.

Inter-observer agreement on lesion detection in the same location was assessed by the index of specific agreement (ISA), defined as the conditional probability that an independent reader detects a lesion in the same location as a randomly selected reader [40, 41]. Inference for ISA was made based on the bootstrap resampling procedure and Wald-test as described above.

## SUPPLEMENTARY MATERIALS FIGURES AND TABLES



## Abbreviations

mpMRI: Multiparametric magnetic resonance imaging
PI-RADSv2: Prostate Imaging-Reporting and Data System version 2
CAD: Computer-aided diagnosis
WP: Whole prostate
PZ: Peripheral zone
TZ: Transition zone
PPV: Positive predictive value
ISA: Index of specific agreement
T2W: T2-weighted MRI
DWI: Diffusion-weighted imaging
ADC: Apparent diffusion coefficient
DCE: Dynamic contrast-enhanced MRI
ROC: Receiver operating characteristic
AUC: Area under the ROC curve

## Appendix

**Table:**

	Trial design	Data acquisition (imaging)	Data processing (imaging)	Monitoring	Data acquisition (surgery and histopathology)	Data processing (Histopathology)	Data correlation	Statistics	Manuscript preparation	Manuscript editing
**Sonia Gaur**	X	X	X	X	X	X	X		X	X
**Nathan Lay**	X		X	X			X		X	X
**Stephanie Harmon**	X	X	X	X		X	X	X	X	X
**Sreya Doddakashi**			X				X	X	X	X
**Sherif Mehralivand**		X		X			X		X	X
**Burak Argun**		X	X		X				X	X
**Tristan Barrett**		X	X		X				X	X
**Sandra Bednarova**		X	X		X				X	X
**Rossanno Girometti**		X	X		X				X	X
**Ercan Karaarslan**		X	X		X				X	X
**Ali Riza Kural**		X	X		X				X	X
**Aytekin Oto**		X	X		X				X	X
**Andrei S. Purysko**		X	X		X				X	X
**Tatjana Antic**					X	X			X	X
**Cristina Magi-Galluzzi**					X	X			X	X
**Yesim Saglican**					X	X			X	X
**Stefano Sioletic**					X	X			X	X
**Anne Y. Warren**					X	X			X	X
**Leonardo Bittencourt**		X	X						X	X
**Jurgen J. Fütterer**		X	X						X	X
**Rajan T. Gupta**		X	X						X	X
**Ismail Kabakus**		X	X						X	X
**Yan Mee Law**		X	X						X	X
**Daniel Margolis**		X	X						X	X
**Haytham Shebel**		X	X						X	X
**Antonio C. Westphalen**		X	X						X	X
**Bradford J. Wood**				X					X	X
**Peter A. Pinto**				X					X	X
**Joanna H. Shih**	X			X			X	X	X	X
**Peter L. Choyke**	X	X	X	X	X	X	X		X	X
**Ronald M. Summers**	X		X	X			X		X	X
**Baris Turkbey**	X	X	X	X	X	X	X		X	X
