# Supplementary material for: Can computer-aided diagnosis assist in the identification of prostate cancer on prostate MRI? a multi-center, multi-reader investigation
Source: Oncotarget. 2018 Sep 18;9(73):33804–17. doi: 10.18632/oncotarget.26100 (PMC6173466; doi:10.18632/oncotarget.26100)
Supplement: Supplementary file 1 [file oncotarget-09-33804-s001.pdf]

Can computer-aided diagnosis assist in the identification of prostate cancer on prostate MRI? a multi-center, multi-reader investigation

SUPPLEMENTARY MATERIALS

Image quality assessment

A critical assumption in the implementation of this CAD system was that the prostate volume segmentation derived for CAD use on T2W imaging could be applied/ transferred to ADC and b-1500 diffusion weighted sequences without registration error (see Methods section Computer Aided Diagnosis Software). However, several studies have demonstrated the impact of gas distortion and patient motion on prostate mpMRI image quality (1-3). Error in prostate segmentation has been shown to be dependent on these image quality factors (4).

To investigate the potential impact of image quality on CAD performance, imaging from all patients was visually evaluated for quality on the basis of image distortion and motion artifacts. Gas-related distortion was evaluated by visual assessment of the amount of gas or stool within the rectum visible on all imaging sequences, qualitatively scored on a scale of 0-2 from none to moderate/severe. Similarly, patient motion was evaluated visually by the appearance of blurring, ghosting, or movement between slices on all imaging sequences and was qualitatively assigned a score of 0 (not-present) or 1 (present). Poor image quality was defined as patients either with motion or distortion artifacts that resulted in T2W prostate segmentation not encompassing the entire prostate volume on diffusion sequences. All scoring was completed by a clinical research fellow with verification/ consensus by an experienced genitourinary radiologist with >10 years experience.

Of 216 patients, 53 were observed to have motion-related artifacts of which 25 had poor concordance between T2W prostate segmentation and prostate volume on diffusion imaging. Gas-related distortion was commonly observed in this patient population, with 64 patients observed as having minimal gas/stool in the rectum and 78 patients with moderate/severe amounts of gas/stool in the rectum. Of patients with some level of gas-related distortion (minimal or moderate/severe), 38 had poor overlap in T2W prostate segmentation and prostate volume on diffusion imaging. The final subset of patients with poor image quality, defined as either motion or gas-related distortion resulting in poor prostate volume concordance, was 52/216 patients.

REFERENCES

1. Caglic I, Hansen NL, Slough RA, Patterson AJ, Barrett T. Evaluating the effect of rectal distension on prostate multiparametric MRI image quality. Eur J Radiol. 2017; 90:174-80.
2. Rosenkrantz AB, Oei M, Babb JS, Niver BE, Taouli B. Diffusion-weighted imaging of the abdomen at 3.0 Tesla: image quality and apparent diffusion coefficient reproducibility compared with 1.5 Tesla. J Magn Reson Imaging. 2011; 33:128-35.
3. Rosenkrantz AB, Taneja SS. Radiologist, be aware: ten pitfalls that confound the interpretation of multiparametric prostate MRI. AJR Am J Roentgenol. 2014; 202:109-20.
4. Gill AB, Czarniecki M, Gallagher FA, Barrett T. A method for mapping and quantifying whole organ diffusion-weighted image distortion in MR imaging of the prostate. Sci Rep. 2017; 7:12727.

Supplementary Table 1: mpMRI acquisition parameters at 3T for institution #1

Parameter	T2 Weighted	DWI*†	DCE MR Imaging‡
Field of view (mm)	180x180	200x200	200x200
Acquisition Matrix	300x291	100x98	168x166
Repetition time (msec)	4656	4458	3.4
Echo time (msec)	80	73	1.75
Flip angle (degrees)	90	90	15
Section thickness (mm), no gaps	3	3	4
Image reconstruction matrix (pixels)	400x400	144x144	320x320
Reconstruction voxel imaging resolution (mm/pixel)	0.45x0.45	1.39x1.39	0.63x0.63
Time for acquisition (min:sec)	5 min 21 sec	7 min 04 sec	6 min 04 sec

Institution #1 utilized a 32-channel surface coil for acquisition. Controls received a 12-core systematic biopsy.
* *ADC maps calculated from evenly spaced b-values 0-1000-1500-2000*
† ***Maximum b-value acquired: 2000***
‡DCE Images obtained, before, during, and after a single dose of Gadobenate dimeglumine 0.1 mmol/Kg at 3 ml/sec. Each sequence obtained at 8.1 s intervals
MRI manufacturer: Phillips

Supplementary Table 2: mpMRI acquisition parameters at 3T for institution #2

Parameter	T2 Weighted	DWI*	High *b*-Value DWI†	DCE MR Imaging‡
Field of view (mm)	220 x 220	280 x 280	240 x 120	220 x 220
Acquisition Matrix	384 x 288	128 x 128	160 x 80	192 x 160
Repetition time (msec)	3712	3775	4000	4.1
Echo time (msec)	102	70	80	1.8
Flip angle (degrees)	111	90	90	17
Section thickness (mm), no gaps	3 / 0 mm	3 / 0 mm	3 / 0 mm	3 / 0 mm
Image reconstruction matrix (pixels)	512 x 512	256 x 256	256 x 256	256 x 256
Reconstruction voxel imaging resolution (mm/pixel)	0.57 x 0.8	1.9 x 1.9	1.5 x 1.5	1.46 x 1.38
Time for acquisition (min:sec)	4:39	2:58	4:52	5:57

Institution #2 utilized a 32-channel phased-array body coil; Cardiac Array by Invivo for acquisition. Controls received a 24 core transperineal template biopsy.
* *ADC maps automatically generated from b-values 150, 750, 1400*
† ***Max b-value acquired: 2000 (separate series, small FOV)***
‡DCE Images obtained, before, during, and after a single dose of Gadobutrol (Gadovist) injection at 28s 3 mL/s (dose 0.1 mmol/kg). Each sequence obtained at *7* s intervals
MRI manufacturer: GE

Supplementary Table 3: mpMRI acquisition parameters at 3T for institution #3

Parameter	SAG T2	COR T2	AX T2	T2 Weighted (SPACE)	DWI*	High *b*-Value DWI†	DCE MR Imaging‡
Field of view (mm)	210	210	180	240	200	Calculated 1500	260
Acquisition Matrix	269 x 384	269 x 384	256 x 320	228 x 320	111 x 130		166 x 256
Repetition time (msec)	3340	3260	3730	2500	4700		3.29
Echo time (msec)	116	126	121	225	86		1.26
Flip angle (degrees)	120	120	138	variable	90		15
Section thickness (mm), no gaps	3	3	3	1	3		3
Image reconstruction matrix (pixels)	.5 x .5 x 3	.5 x .5 x 3	.6 x .6 x 3	.8 x .8 x 1	1.5 x 1.5 x 3		.1 x .1 x 3
Reconstruction voxel imaging resolution (mm/pixel)				.75 x .75 x 1.5			1.56 x 1.02 x 3.86
Time for acquisition (min:sec)	1:55	2:31	2:53	6:04	5:07		4:41

Institution #3 utilized a 18 channel phased array body coil and 32 channel Spine Coil for acquisition. Controls received a systematic 12-core TRUS-guided biopsy.

* *ADC maps calculated from 3 evenly spaced b values (50, 400, 900)*

† ***Max acquired b-value:*** 900 (calculated 1500)

‡DCE Images obtained, before, during, and after a single dose of Dotarem (Gd) 0.2 mL/kg (0.1 mmol/kg) body weight at 2cc/sec followed by 2cc/sec of saline (20ml). Each sequence obtained at 7sec intervals

MRI manufacturer: Siemens

Supplementary Table 4: mpMRI acquisition parameters at 3T for institution #4

Parameter	T2 Weighted	DWI*	High *b*-Value DWI†	DCE MR Imaging‡
Field of view (mm)	160x160	180x180	180x180	220x258
Acquisition Matrix (mm)	0.4x0.4	1.5x1.5	1.5x1.5	1.5x1.5
Repetition time (msec)	5709	3733-5000	3733-5000	5
Echo time (msec)	200	57, 70,150,200	57, 70,150,200	1.5/2.8
Flip angle (degrees)	90	90	90	10
Section thickness (mm), no gaps	3	3	3	3
Image reconstruction matrix (pixels)	512x512	128x128	128x128	320x320
Reconstruction voxel imaging resolution (mm/pixel)	0.31x0.31x3	1.4x1.4x3	1.4x1.4x3	0.8x0.8x1.5
Time for acquisition (min:sec)	3min59.8sec	1:45 -3:30min	1:45 -3:30min	7:30min

Institution #4 utilized a 16 Channel anterior and posterior coil for acquisition.

* *4 b-value DWI images used to fit a mono-exponential decay model to calculate ADC maps. The b-values are:0, 150, 1000, 1500*

† ***Maximum acquired b=1500***

‡DCE Images obtained, before, during, and after a dose of MultiHance at body weight(kg)x0.2ml. Each sequence obtained at 7.5 s intervals, DCE sequence has total 60 dynamic scans, 4 scans are before injection, the injection was performed at the 5th dynamic scan.

MRI manufacturer: Phillips

Supplementary Table 5: mpMRI acquisition parameters at 3T for institution #5

Parameter	T2 Weighted	DWI*	High *b*-Value DWI†	DCE MR Imaging
Field of view (mm)	210x210	250x250	calculated 1500	300x250
Acquisition Matrix	256x320	120x160		158x256
Repetition time (msec)	3000	3300		8
Echo time (msec)	100	60		2.5
Flip angle (degrees)	120	90		20
Section thickness (mm), no gaps	3.5	3.5		3
Image reconstruction matrix (pixels)	320x320	160x160		256x256
Reconstruction voxel imaging resolution (mm/pixel)	0.68x0.68x3.5	1.6x1.6x3.5		1.2x1.2x3
Time for acquisition (min:sec)	2:06	5:00		4:52

Institution #5 utilized a surface array coil for acquisition.

† ***max b-value provided = 800***

Supplementary Table 6: Tumor-based CAD training population. All patients imaged at 3T without endorectal coil, with no institutional or acquisition overlap with study population

	Gleason score					
	**Control**	**6**	**3+4**	**4+3**	**8**	**9**
Number of tumors (patients for control)	25	25	35	19	12	4

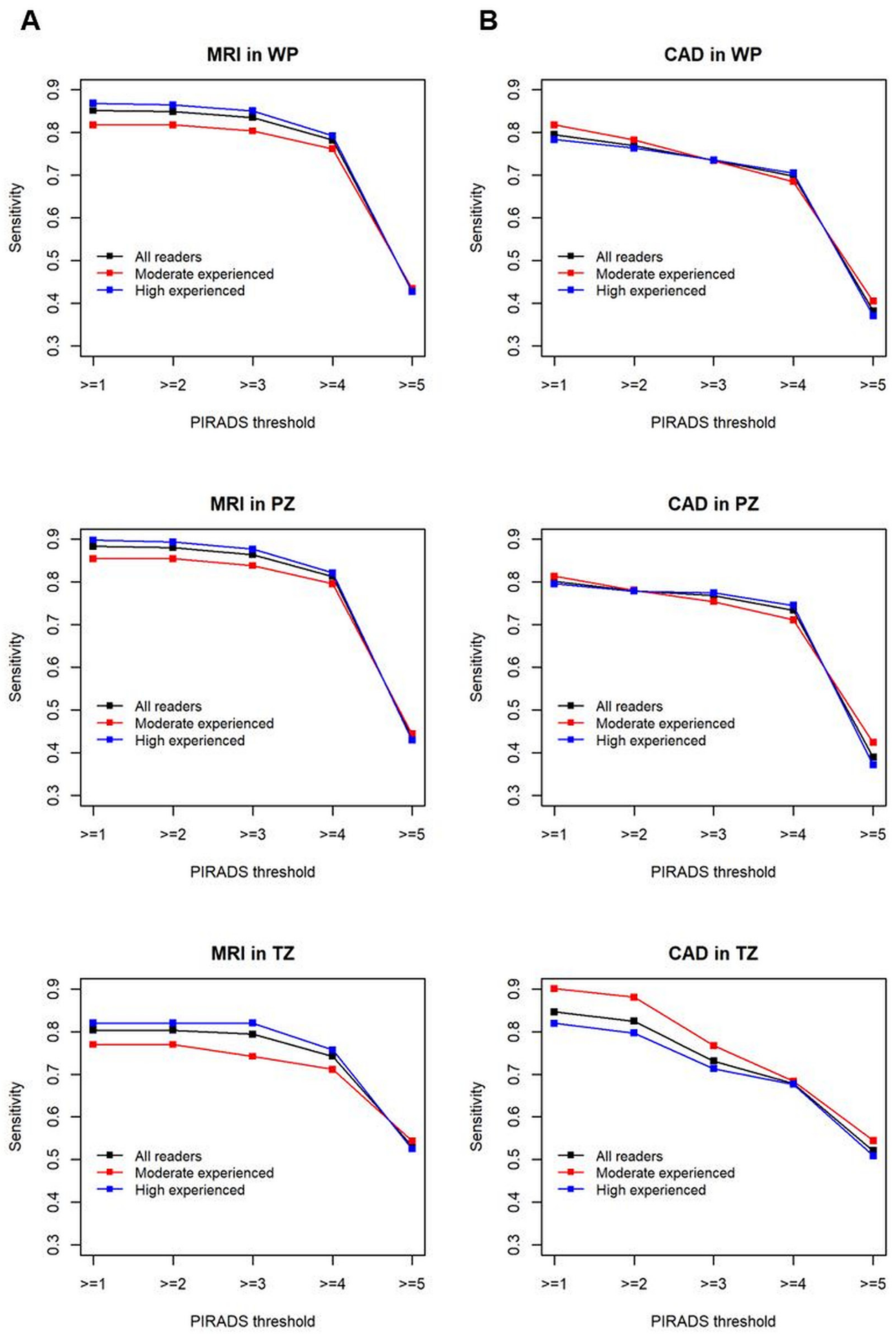

Supplementary Figure 1: Clinically significant index lesion sensitivity in WP, PZ, TZ for MRI-only **(A)** and CAD-assisted **(B)** reads. Sensitivities are plotted for all readers as well as by experience level at each PI-RADSv2 category threshold. PI-RADSv2 category ≥1 threshold used for all lesions detected on MRI and CAD, while PI-RADSv2 category ≥3 threshold used to represent all lesions considered cumulatively suspicious on MRI and CAD. Clinically significant was defined as Gleason score ≥7, WP = whole prostate, PZ = peripheral zone, TZ = transition zone.

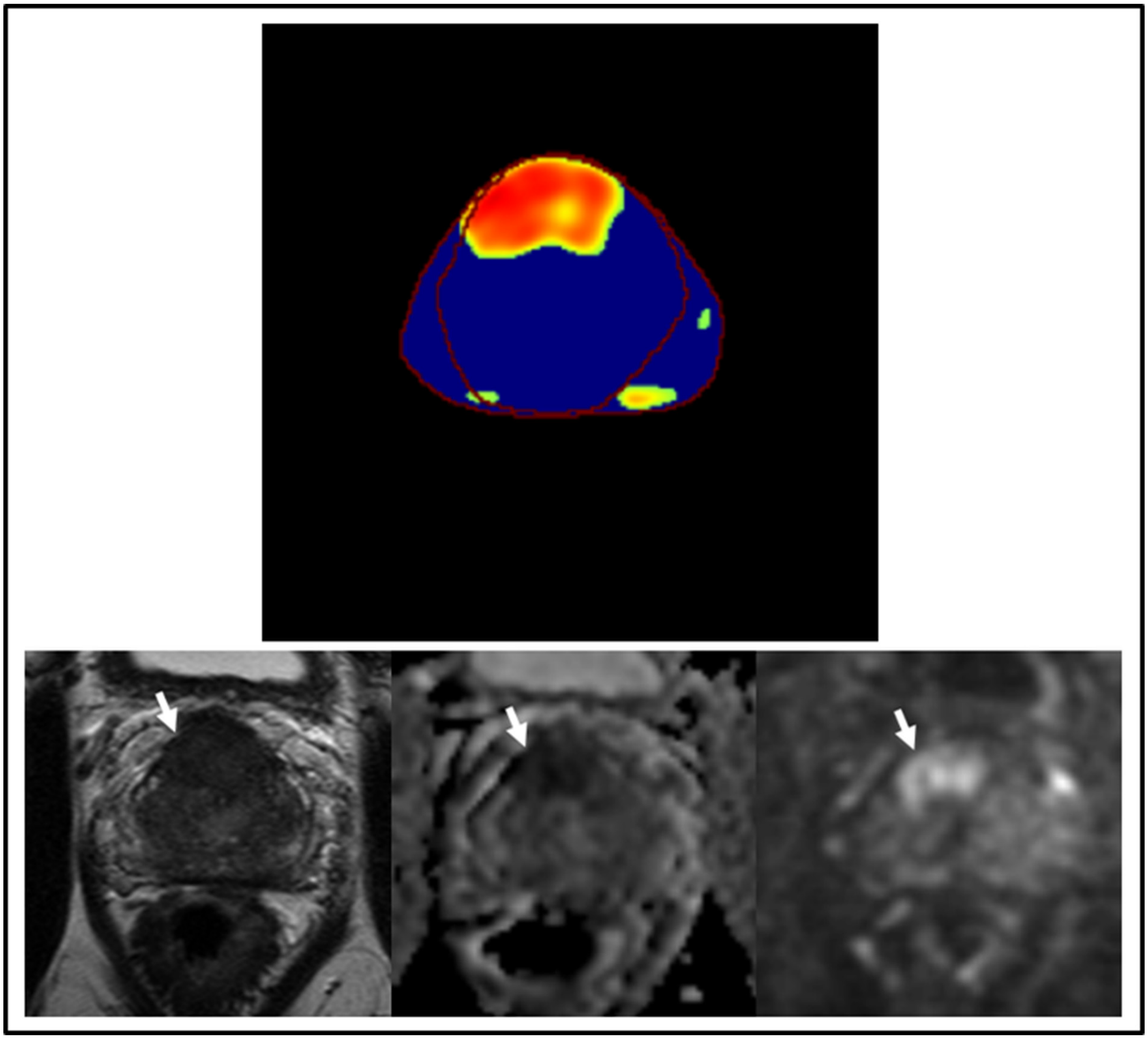

Supplementary Figure 2: Example of CAD output. CAD output mirrors the prostate at each mpMRI slice. Inputs (left to right: T2W, ADC, b-1500) are shown below the resulting output for comparison. The heat map is reflective of probability to represent prostate cancer: red-orange reflects higher likelihood, while yellow-green reflects lower likelihood. Arrows point to the tumor on mpMRI.
